# Short-term outcomes of switching anti-VEGF agents in eyes with treatment-resistant wet AMD

**DOI:** 10.1186/s12886-015-0025-z

**Published:** 2015-04-11

**Authors:** Figen Batioglu, Sibel Demirel, Emin Özmert, Ahmet Abdullayev, Serdar Bilici

**Affiliations:** Department of Ophthalmology, Ankara University Medical Faculty, Mamak caddesi, Vehbi Koç Göz Hastanesi, Dikimevi, Ankara, Turkey

**Keywords:** Aflibercept, Age-related macular degeneration, Ranibizumab

## Abstract

**Background:**

To investigate the short-term outcomes of treatment with intravitreal aflibercept in cases with wet age-related macular degeneration (AMD) resistant to ranibizumab.

**Methods:**

The study included patients who had been undergoing follow-up for a minimum of three months at the Ankara University Faculty of Medicine Ophthalmology Department’s Retina Unit with a diagnosis of wet AMD. All cases had received intravitreal aflibercept injection due to the presence of intraretinal/subretinal fluid and pigment epithelial detachment (PED), as detected by optical coherence tomography (OCT), despite having received intravitreal ranibizumab. Medical records of the cases were investigated retrospectively and the demographic data, treatments administered before aflibercept injection, best-corrected visual acuity (BCVA) before and after aflibercept injection, central macular thickness (CMT), and the presence of intraretinal/subretinal fluid and the height and presence of PED were recorded.

**Results:**

A total of 29 eyes from 11 females and 17 males were included in the study. The mean age was 73.89 ± 7.49 (62–92). The average number of intraocular injections administered before aflibercept injection was 11.75 ± 5.73 (6–25). The mean duration of follow-up following aflibercept injection was 4.55 ± 2.14 (3–11) months, with a mean of 3.44 ± 0.73 (3–5) aflibercept injections during this period. The mean BCVA values before and after aflibercept injection were found to be 0.83 and 0.77 LogMAR, respectively. The mean CMT values before and after aflibercept injection were 471.3 (97–1365) and 345.1 (97–585) microns, respectively (p < 0.001). The PED height before and after aflibercept injection was 350.4 ± 151.7 (129–793) and 255.52 ± 156.8 (0–528) microns, respectively (p < 0.05).

**Conclusion:**

Switching to intravitreal aflibercept appears to be an effective treatment modality for patients with AMD who are resistant to ranibizumab. While anatomic success including the effect of reducing the PED height was achieved in the short term following aflibercept injection in all cases, no concomitant increase in visual acuity occurred. This is attributed to the long-term presence of chronic fluid and the development of scar tissue before the treatment.

## Background

Multicenter trials have demonstrated the efficacy and safety of vascular endothelial growth factor (VEGF) inhibitors in the treatment of wet age-related macular degeneration (AMD) [1-8]. It has been reported that visual acuity was maintained in 90% of the patients and there was increased visual acuity in approximately 30% of the patients receiving ranibizumab when it was administered monthly or “as required” (pro re nata) [6-8]. Despite the favorable results reported in multicenter studies, AMD is a chronic disorder that requires continuous follow-up and treatment. Based on the SEVEN-UP study reporting the 7-year follow-up results of 65 wet AMD cases that were receiving ranibizumab treatment and participated in the ANCHOR, MARINA, and HORIZON studies, 50% of the patients required active treatment at the end of the 7th year [9]. This may be the result of reactivations related to the natural course of the disease or due to the occurrence of tachyphylaxis or tolerance to treatment associated with long-term intravitreal drug use [10,11]. In such cases, use of different treatment agents is considered.

Aflibercept is a recombinant soluble decoy receptor that is composed of components of both VEGF receptor 1 (VEGFR1) and VEGF receptor 2 (VEGFR2) fused to the Fc region of human IgG1. This molecule has a higher affinity for binding to VEGF-A, a protein to which ranibizumab and bevacizumab also bind, and it also inhibits VEGF-B and placental growth factor (PIGF) [12-16]. Based on the results from the VIEW 1 and 2 studies, aflibercept has been approved by the FDA for treatment of wet AMD [17]. These multicenter, randomized, double-blind studies have demonstrated that anatomic and visual outcomes with 2 mg aflibercept injections administered every 8 weeks following a 3-month loading dose were comparable to those obtained with monthly ranibizumab injections. Subsequently, it has been put to use as a first choice of treatment or in cases resistant to ranibizumab injection.

In this study, we assessed the short-term anatomic and visual outcomes of intravitreal aflibercept in cases with wet AMD resistant to intravitreal ranibizumab.

## Methods

The study included patients who had been on long-term ranibizumab for the treatment of wet AMD and had switched to intravitreal aflibercept injection. Inclusion criteria were as follows: persistent intraretinal or subretinal fluid with or without PED, at least six consecutive monthly injections with ranibizumab, and last injection of ranibizumab within 28–35 days of switching to aflibercept. The exclusion criteria included a history of intraocular surgery, except for uncomplicated phacoemulsification performed within the preceding 6 months; history of subfoveal laser photocoagulation; uncontrolled glaucoma or uveitis; and any ocular disease that could affect the BCVA in the study eye. The study was conducted in accordance with the Declaration of Helsinki and written informed consent for participation in the study was obtained from participants. The local ethics committee approval for human subjects research was received from Ankara University Medical Faculty with study protocol 20-848-14.

All patients received a loading dose of three monthly aflibercept injections (2 mg/0.05 ml) and were followed up monthly. Retreatment with a single aflibercept injection was performed according to any of the following: visual acuity loss of at least five letters, with optical coherence tomography (OCT) evidence of fluid in the macula; persistent or recurrent intraretinal or subretinal fluid on OCT; new subretinal hemorrhage from choroidal neovascularization (CNV).

Before and after aflibercept initiation, all patients had a complete ophthalmic examination including BCVA measurement, OCT, and fundus photography. Best-corrected visual acuity was measured using the standardized, 70-letter Early Treatment Diabetic Retinopathy Study (ETDRS) chart at 4 meters distance. Optical coherence tomography images were obtained using the Spectralis OCT (Spectralis Heidelberg Engineering, Heidelberg, Germany) following a standardized protocol. The medical records of patients with a minimum 3-month follow-up duration since transition to aflibercept treatment were retrospectively investigated. The demographic data, the treatments administered before aflibercept injection, the BCVA before and after aflibercept injection, the central macular thickness (CMT), the presence of intraretinal/subretinal fluid, and the height and presence of PED detected by OCT were recorded. Changes in intraretinal/subretinal fluid or PED height were described as complete/partial resolution or stable/worsened, similar to descriptions in previously published studies that discussed switching anti-VEGF treatment [10,18]. In addition, potential side effects were investigated, including endophthalmitis following injection, intraocular pressure increase requiring medical treatment, retinal detachment, uveitis, and thromboembolic events.

## Results

The study included 29 eyes of 28 patients receiving intravitreal 2 mg/0.05 ml aflibercept injection for the treatment of wet AMD resistant to intravitreal ranibizumab injections. There were 11 females and 17 males with a mean age of 73.89 ± 7.49 (62–92). The average number of intra-ocular injections administered before aflibercept injection was 11.75 ± 5.73 (6–25). Prior to ranibizumab treatment, two cases received 2 and 4 intravitreal bevacizumab injections, respectively, while another one case was administered photodynamic therapy once and intravitreal pegaptanib three times. All patients who switched to aflibercept treatment had ongoing intraretinal and/or subretinal fluid with or without PED despite previous therapies. The mean duration of follow-up following aflibercept injection was 4.55 ± 2.14 (3–11) months, with cases receiving an average of 3.44 ± 0.73 (3–5) aflibercept injections during this period (Table 1).

**Table 1:** **Clinical characteristics of patients switched to aflibercept from ranibizumab**

Average number of intraocular injections before	11.75±5.73 (6-25).
Aflibercept injection (average-interval)	3.44±0.73 (3-5)
BCVA values (average /logMAR-ETDRS letters)	
Before aflibercept injection	1.08 (47.4 letters)
After aflibercept injection	0.80 (50 letters)
Central macular thickness (average-interval)	
Before aflibercept injection	471.3 (97-1365)
After aflibercept injection	345.1 (97-585)
The mean height of PED	
Before aflibercept injection	350.4 ± 151.7 (129-793)
After aflibercept injection	255.52 ± 156.8 (0-528)

BCVA: Best corrected visual acuity.

PED: Pigment epithelial detachment.

The mean BCVA values before and after aflibercept injection were 0.83 (44 ETDRS letters) and 0.77 LogMAR (48 ETDRS letters), respectively. The mean CMT values before and after aflibercept injection were 471.3 (97–1365) and 345.1 (97–585) microns, respectively (p < 0.001). The PED height before and after aflibercept injection was 350.4 ± 151.7 (129–793), and 255.52 ± 156.8 (0–528) microns, respectively (p < 0.05) (Table 1).

**Figure 1:**
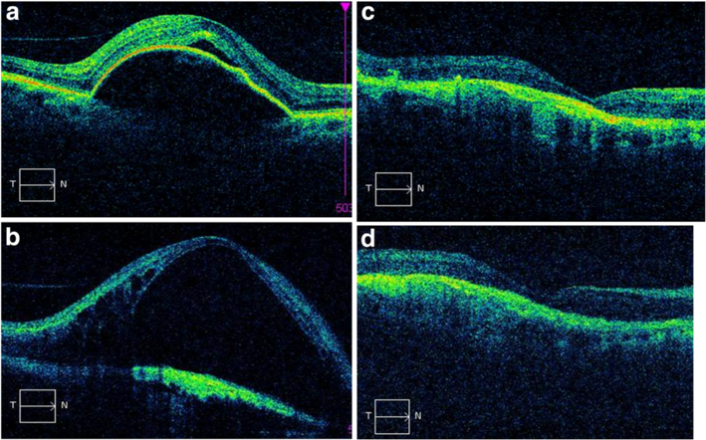
Good anatomical response to 3 aflibercept injections in a ranibizumab resistant AMD case. **a**. PED before intravitreal ranibizumab (VA: 0.8 LogMAR). **b**: Intense subretinal fluid is observed on OCT despite 24 ranibizumab injections (VA: 2.1 LogMAR). **c**. Subretinal fluid has completely disappeared after a single aflibercept injection on OCT (VA: 2.1 LogMAR). **d**. No subretinal fluid is seen after the third aflibercept injection on OCT (VA: 2.1 LogMAR)

**Figure 2:**
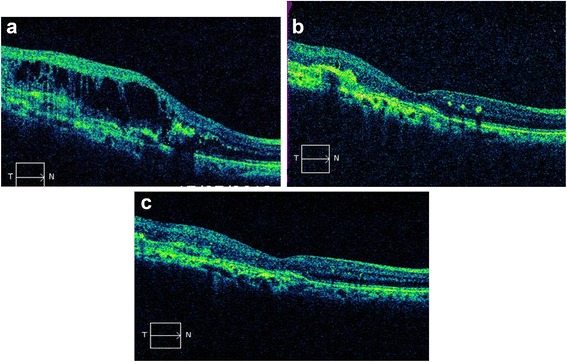
Visual and anatomical improvement after 3 aflibercept injections in a ranibizumab resistant AMD case. **a**. Large-septa cysts are seen on OCT following 11 ranibizumab injections (VA: 1.3 LogMAR). **b**: The cysts have completely disappeared after a single aflibercept injection (VA: 0.7 LogMAR). **c**. There is no cyst on OCT and VA has been increased after the third aflibercept injection (VA:0.4 LogMAR). AMD: Age-related macular degeneration. PED: Pigment epithelial detachment. VA: Visual acuity. OCT: Optic coherence tomography.

Before switching to aflibercept treatment, 5 eyes (17.24%) had intraretinal/subretinal fluid and 24 eyes (82.76%) had intraretinal/subretinal fluid and PED (22 fibrovascular, 2 serous). Following aflibercept injections, of the 29 eyes with intraretinal/subretinal fluid, 17 eyes (58.6%) had complete resolution, 10 eyes (34.4%) had partial improvement, and 2 eyes (6.8%) remained stable. On the other hand, of the 24 eyes with PED, 2 eyes (8.3%) (1 serous, 1 fibrovascular) had complete resolution, 16 eyes (66.6%) had partial improvement of PED, 4 eyes (16.6%) remained stable, while 2 cases (8.3%) had an increase in PED height (Table 2) (Figures 1a–c, 2a,b). None of the patients experienced any of the potential side effects that could occur following intraocular injection, such as endophthalmitis, increased intraocular pressure requiring medical treatment, retinal detachment, uveitis, or thromboembolic events.

**Table 2:** **Spectral-domain optical coherence tomography analysis before and after aflibercept injection**

***SD-OCT*** **segmentation**	***Before aflibercept***	***Complete resolution***	***Partial resolution***	***Stable***	***Deterioration***
Intraretinal/subretinal fluid	29	17	10	2	-
*without PED*	*5*	*2*	*3*	*-*	*-*
*with PED*	*24*	*15*	*7*	*2*	*-*
PED	24	2	16	4	2

SD-OCT: Spectral domain optic coherence tomography.

PED: Pigment epithelial detachment.

## Discussion

Most patients with wet AMD require repeated intravitreal injections in the long term [9]. This may be a result of the ongoing activity of the disease or due to the occurrence of tachyphylaxis or tolerance to the medication. Tachyphylaxis is the lack of response that occurs when a drug is used at short intervals and no response can be achieved upon dose escalation. However, when treatment is interrupted for a short time, the efficacy of the drug is regained [19]. In contrast, tolerance is a reduction in the extent and duration of a drug’s efficacy over time as a result of long-term use. The efficacy of a drug may be improved when the dose is increased or is administered at shorter intervals. However, temporary interruption of the treatment does not lead to an improvement [19]. Development of tachyphylaxis in some patients receiving repeated ranibizumab/bevacizumab injections has been reported in several trials [10,11]. Gasperini et al. showed that 81% of the tachyphylactic patients in their study responded favorably after switching from either ranibizumab to bevacizumab or vice versa [10]. With systemic and local immune response occurring in the cases following intravitreal injection, neutralizing antibodies may develop against bevacizumab/ranibizumab. In addition, several authors have suggested that VEGF production could be upregulated via macrophages in choroidal neovascular tissue due to chronic VEGF blockage [11,20-22].

In the current study, we evaluated the functional and anatomic response of patients with refractory neovascular AMD after conversion to aflibercept injection. The lack of visual improvement in the eyes affected with recalcitrant neovascular AMD may be related to longstanding retinal damage as a result of persistent intraretinal and subretinal exudation. There is growing evidence that resistant eyes respond well to different anti-VEGF agents in some studies in which both functional and anatomical improvement occurred within the first few months. Aflibercept is a novel inhibitor that provides an alternative VEGF blockage. After the efficacy of intravitreal aflibercept in wet AMD cases was demonstrated, it was introduced for use in patients who are resistant to other anti-VEGF agents. The current relevant studies are summarized in Table 3. These published studies have reported anatomic improvement [18,23-28], in particular. In one of these studies, Yonekawa et al. analyzed the outcomes of 102 eyes of 96 patients with chronic neovascular AMD and reported that replacing ranibizumab with aflibercept in patients with chronic neovascular AMD resulted in stabilized vision and improved anatomic outcomes. After the switch, 91% of the eyes with refractory AMD showed improvement in OCT findings, while 9% were stable and no eyes worsened. In line with these results, our study also particularly revealed anatomic improvement following aflibercept injections. According to OCT findings, among the 29 eyes with baseline intraretinal/subretinal fluid, 17 (58.6%) had complete resolution, 10 (34.4%) had partial resolution, 2 (6.8%) remained stable, and none had worsened. The pre-treatment mean CMT value of 471.3 microns was decreased to 345.1 microns after the treatment.

**Table 3:** **Summary of the similar studies on aflibercept treatment in resistant AMD cases**

**Study group**	**No of eyes**	**Mean age**	**No of previous injections**	**No of Afl injections**	**Mean follow-up time (month)**	***Visual acuity (LogMAR)***		**CMT (micron)**	
						**Before Afl**	**After Afl**	**Before Afl**	**After Afl**
Cho et al.^1^	28	80,68 (62-95)	20,2±7,6 (7-37)	4,4 (3-6)	171 (134–192) days	0,52	0,57	295	274
Bakall et al.^2^	36	79 (60-88)	25,6±(6-74)	5,2 (4-6)	6	0,45	0,50	410 (174-1027)	296 (151-528)
Ho et al ^3^	96	79 (62-91)	17 (1-60)	2,6 (2-4)	114 (90-133) days	20/50 (snellen)	0,02 (LogMAR)	276 (130-559)	258 (198-242)
Yonekawa et al ^4^	102	79,6 (57-93)	20,4 (3-65)	3,8 (1-8)	18,4 weeks	0,42	0,38	305,07	276,20
Kumar et al ^5^	34	79 (72-84)	28,6 (10-47)	5,3 (5-6)	6,5 (6-6,6)	0,57	0,47	416±217	348±171
Hall et al ^6^	30	80,4 (-)	14,9 (2-53)	6,27 (4-11)	10,4 (3-12)	0,533*	0,521*	264±12,5*	237±10,2*
Heussen et al ^7^	71	77 (43-95)	9 (3-43)	2,7 (1-4)	-	0,56^+^	0,43^+^	350.8±115^+^	260.8±34^+^

*values of 22 eyes with 12 moths follow-up period.

^+^values of 45 eyes which had 3 aflibercept injections.

Afl: Aflibercept, CMT: Central macular thickness.

^1^ *Cho H, Shah CP, Weber M, Heier JS. Aflibercept for exudative AMD with persistent fluid on ranibizumab and/or bevacizumab. Br J Ophthalmol 2013;97(8):1032-5.*

^2^ *Bakall B, Folk JC, Boldt HC, et al. Aflibercept therapy for exudative age-related macular degeneration resistant to bevacizumab and ranibizumab. Am J Ophthalmol. 2013;156(1):15-22.*

^3^ *Ho VY, Yeh S,olsen TW, et al. Short-term outcomes of aflibercept for neovascular age-related macular degeneration in eyes previously treated with other vascular endothelial growth factor ınhibitors. Am J Ophthalmol. 2013;156(1):23-28.*

^4^ *Yonekawa Y, Andreoli C, Miller JB, et al. Conversion to aflibercept for chronic refractory or recurrent neovascular age-related macular degeneration. Am J Ophthalmol. 2013;156(1):29-35.*

^5^ *Kumar N, Marsiglia M, Mrejen S, et al. Visual and anatomical outcomes of intravitreal aflibercept in eyes with persistent subfoveal fluid despite previous treatments with ranibizumab in patients with neovascular age-related macular degeneration. Retina 2012;33:1605–1612.*

^6^ *Hall LB, Zebardast N, Huang JJ and Adelman RA. Aflibercept in the treatment of neovascular age-related Macular degeneration in previously treated patients. J Ocul Pharmacol Ther. DOI: 10.1089/jop.2013.0188*.

^7^ *Heussen FM, Shao Q, Ouyang Y et al. Clinical outcomes after switching treatment from intravitreal ranibizumab to aflibercept in neovascular age-related macular degeneration. Graefes Arch Clin Exp Ophthalmol DOI 10.1007/s00417-013-2553-7.*

Various treatments of PED have been attempted; however, none has been demonstrated to be completely effective. Recently, association of PED type and response to anti-VEGF treatment were investigated [29,30]. According to Hoerster et al., fibrovascular PEDs were resistant to treatment with ranibizumab, while serous PEDs responded well [29]. Inoue et al. also revealed that 100% of serous and mixed PEDs showed reduction in PED height by 100%, while this ratio was remained at 67% in fibrovascular PED [30]. In the current study, there was a statistically significant decrease in the mean PED height (from 350.4 to 255.52 microns, p < 0.05). While 16 of 24 (66.6%) eyes with PED (15 fibrovascular 1 serous PED) showed partial improvement in PED height, 2 (8.3%) eyes with PED (1 serous, 1 fibrovascular) showed complete resolution after aflibercept treatment. There was a 27.05% decrease in the mean PED height after switching to aflibercept treatment.

Basically, there are two mechanisms may be involved in the improvement achieved in anatomic outcomes with aflibercept in patients with wet AMD resistant to other anti-VEGF treatments. One is the fact that this molecule has a stronger and wider spectrum of efficacy. While bevacizumab and ranibizumab are human IgG1 isotypes, aflibercept is a monoclonal antibody with a 100-fold higher VEGF-A binding affinity, which is obtained by the integration of VEGF receptors 1 and 2, and IgG-1 crystalline fragments. In addition to this high binding affinity, aflibercept also inhibits other factors that affect neovascularization, such as VEGF-B and placental growth factor [12-16]. The second; studies suggest that patients treated with repeated intravitreal injections of bevacizumab or ranibizumab may have developed an immunity [10,11]. By switching to aflibercept, lesser immunogenicity associated with a new agent theoretically may lead to sustained anatomic outcomes in eyes refractory to ranibizumab [19]. In the similar studies, the anatomical results exceed the functional results [18,23-28]. Heussen et al. analyzed the clinical outcomes of 71 eyes of 65 AMD patients after transition to aflibercept treatment. They reported that 33% of cases with no functional improvement under ranibizumab therapy gained visual acuity after transition to aflibercept [28]. In the current study, before switching to aflibercept treatment, eyes had persistent fluid despite 11.75 ?>(6–25) intraocular ranibizumab injections on average. The visual potential after switching to aflibercept might have been limited by the development of chronic photoreceptor degeneration. Thus, visual improvement in addition to anatomic improvement may be achieved by switching to aflibercept treatment earlier in patients who are considered resistant to ranibizumab treatment.

## Conclusions

The current study presents significant results on the efficacy of aflibercept in the treatment of patients with wet AMD resistant to ranibizumab. Intravitreal aflibercept injections represent a strong alternative for treatment-resistant cases. However, the retrospective design and the limited number of cases constitute limitations of this study. Larger case series need to be investigated prospectively and with a long-term follow-up for more enlightened and long-term results to be obtained.
